# Evaluating the performance of temporal pattern discovery: new application using statins and rhabdomyolysis in OMOP databases

**DOI:** 10.1186/s12911-022-01765-1

**Published:** 2022-02-03

**Authors:** M. Lavallee, T. Yu, L. Evans, M. Van Hemelrijck, C. Bosco, A. Golozar, A. Asiimwe

**Affiliations:** 1 Former Bayer Healthcare Pharmaceutical Inc, Whippany, NJ USA; 2 Virginia Commonwealth University, Richmond, VA USA; 3 LTS Computing LLC, West Chester, PA USA; 4 Translational Oncology & Urology Research (TOUR), King’s College London, London, UK; 5 Bayer AG, Berlin, Germany

**Keywords:** Temporal pattern discover, Adverse events, Statins, Rhabdomyolysis

## Abstract

**Background:**

Temporal pattern discovery (TPD) is a method of signal detection using electronic healthcare databases, serving as an alternative to spontaneous reporting of adverse drug events. Here, we aimed to replicate and optimise a TPD approach previously used to assess temporal signals of statins with rhabdomyolysis (in The Health Improvement Network (THIN) database) by using the OHDSI tools designed for OMOP data sources.

**Methods:**

We used data from the Truven MarketScan US Commercial Claims and the Commercial Claims and Encounters (CCAE). Using an extension of the OHDSI ICTemporalPatternDiscovery package, we ran positive and negative controls through four analytical settings and calculated sensitivity, specificity, bias and AUC to assess performance.

**Results:**

Similar to previous findings, we noted an increase in the Information Component (IC) for simvastatin and rhabdomyolysis following initial exposure and throughout the surveillance window. For example, the change in IC was 0.266 for the surveillance period of 1–30 days as compared to the control period of − 180 to − 1 days. Our modification of the existing OHDSI software allowed for faster queries and more efficient generation of chronographs.

**Conclusion:**

Our OMOP replication matched the we can account forwe can account for of the original THIN study, only simvastatin had a signal. The TPD method is a useful signal detection tool that provides a single statistic on temporal association and a graphical depiction of the temporal pattern of the drug outcome combination. It remains unclear if the method works well for rare adverse events, but it has been shown to be a useful risk identification tool for longitudinal observational databases. Future work should compare the performance of TPD with other pharmacoepidemiology methods and mining techniques of signal detection. In addition, it would be worth investigating the relative TPD performance characteristics using a variety of observational data sources.

## Background

The use of healthcare databases as complementary data sources for drug safety signal detection has increasingly been explored as an alternative to spontaneous reporting [1]. This alternative strategy is based on the active collection of information on all adverse events in a cohort exposed to a drug of interest [2]. Hence, information is collected on all patients in the cohort, not just those with suspected adverse drug events. Moreover, all adverse events are recorded and there is a possibility to assess information on adverse events prior to the drug exposure (i.e. a control window for comparison) [2, 3].

The use of prescription-event data in a real-world setting therefore allows for improved spontaneous reports-type analyses because the number of exposed patients is known, more information is available of potential risk factors and confounders, and it may even be possible to retrospectively assess missing information. It also allows the assessment of adverse event profiles by making comparisons with other drugs and across various time periods [2, 3]. Nevertheless, all these potential benefits in terms of data collection strategies also result in complexities of the underlying analytical models.

Signal detection methods to enable identification of risks of medical products in observational healthcare data have hence become more apparent. Leveraging healthcare databases for drug safety signal detection requires evaluation of performance of existing methods to determine which is most appropriate for mining adverse drug reactions. One such method is temporal pattern discovery (TPD), which was originally proposed by Noren et al. in 2010 [4]. The TPD method is appealing because it allows for an open-ended approach to signal detection, applies a shrinkage to prevent spurious signals and uses a self-controlled contrast to identify true temporal associations. It is therefore of interest to also assess whether the TPD method can be used for detecting rare adverse drug events.

Hauben et al. investigated the use of TPD in the context of rare adverse drug events by assessing signals between statin use (simvastatin, atorvastatin, rosuvastatin, fluvastatin, and pravastatin) and development of rhabdomyolysis in the UK primary care database, The Health Improvement Network (THIN) [5]. This TPD application was conducted using the CVW Longitudinal software from Commonwealth Informatics [6], a reimplementation and extension of the vigiTrace software designed for TPD by the Uppsala Monitoring Centre, which is not an open source software. The application of TPD on statins in the THIN database did not detect temporal patterns for statins and rhabdomyolysis, but did detect temporal patterns for Simvastatin and Cerivastatin when the outcome was generalised to “Myalgia and myositis unspecified” [5].

Here, we aimed to replicate the statins study conducted in the THIN database using open-source software from Observational Health Data sciences and Informatics (OHDSI— ICTemporalPatternDiscovery [7]) on data mapped to the OMOP common data model [8]. In addition to ensuring transparency of our TPD statins replication using open source software, conducting the analysis using OMOP data ensures that we can account for differences in results due to database heterogeneity [9] and through consideration of different analytical parameterisations [10]. Our method was also designed so that we can evaluate the operating characteristic of the TPD method. Hence, our study aimed to further develop TPD software as to promote its usefulness for future adverse event reporting studies.

## Methods

### Data source

To replicate and expand the TPD analysis conducted with the THIN data [5], we used data from the Truven Health MarketScan Research Databases capturing private and public claims data in the United States to assess signals between statin use (simvastatin, atorvastatin, rosuvastatin, fluvastatin, and pravastatin) and development of rhabdomyolysis [11]. We used the Commercial Claims and Encounters (CCAE) dataset (n = 138.5 million) and the Medicare Supplemental (MDCR) dataset (n = 9.8 million). These Truven MarketScan datasets allow for real-time analysis of real-world data with clinical details along the complete longitudinal patient record of healthcare encounters and payments. They hence cover the full continuum of healthcare in the US from an administrative claims standpoint. The CCAE and MDCR data were mapped to the OMOP common data model, providing a standardized representation of clinical concepts in a healthcare database [8]. Using OMOP data ensures that the TPD method can be implemented consistently across multiple databases.

### Statistical methods

Our methods are an adaption of the framework for open-ended pattern discovery in large patient record repositories as described by Noren et al. in 2010 [4]. Further details of the underlying statistical code can be found here: https://github.com/OHDSI/TpdChronograph.

#### Noren framework

Temporal pattern discovery (TPD) is an exploratory signal detection framework for longitudinal observational databases. The basis of the method is a graphical statistical approach to highlight and visualise temporal associations between the onset of a prescription drug and the subsequent occurrence of a medical event [4]. The TPD method utilises a calibrated self-controlled cohort design, creating a within cohort adjustment for time-invariant confounders and external cohort contrast to adjust for systematic differences between time periods [2]. Central to the TPD method is the Information Component (IC) measure of disproportionality, which is a transformed ratio of the observed number of events for a drug-outcome combination to the expected number of events, found by the marginal counts. The IC value includes a log base 2 transformation to improve interpretability and a shrinkage of 0.5 to the numerator and denominator to control for volatility of rare events. The IC value is the log posterior mean of the rate of incidence for the drug outcome combination, so that it is possible to construct Bayesian 95% credibility intervals for inference [4].

A graphical tool called the chronograph can then be used as a visualisation for open-ended temporal signal detection. The chronograph is a two-tier chart where the top tier is the IC value and credible intervals plotted temporally indicating variation in the observed-to-expected ratio of events. The bottom tier is a bar chart of the observed number of events overlaid by the line graph of expected events highlighting absolute differences between the observed and expected number of events over time [4].

A summary statistic of the measure of temporal association (IC_Δ_) is calculated by dividing the IC value of a surveillance period (a time window following drug exposure) by the IC value a control period (a time window before drug exposure). The time at risk of both the surveillance window and control window must be specified before calculating the IC_Δ_, thus different parameterisation of the analytical windows will lead to different results. The Bayesian 95% credibility interval may also be calculated for IC_Δ_. Typically, if the lower bound of the credibility interval is greater than 0, IC_Δ025_ > 0 this is considered to be the threshold to identify a temporal association using TPD [2]. The measure of temporal association can be calculated using the OHDSI R package ICTemporalPatternDiscovery.

#### Parameterisation of TPD framework

A flaw in most observational studies is that they do not provide operating characteristics for the system generating evidence. Observational study results are intrinsically impacted by confounding and systematic bias, threatening study validity. A solution to mitigate and visualise issues of systematic bias in observational studies is to evaluate the performance of TPD using positive and negative controls. Emulating an evaluation of drug safety signal detection methods, we ran 60 positive and negative controls through four analytical settings (i.e. different analysis parametrisations) and calculated sensitivity, specificity, bias and area under the curve (AUC) as metrics to assess performance [12]. Positive controls are drug-outcome combinations that have known association, such as statins in this context. Negative controls are drug-outcome combinations with no association known, meaning we would expect the IC_Δ_ measure for each negative control to be around 0 in a TPD analysis.

Following this evaluation, we selected the analytical setting for determining a temporal association between statin-rhabdomyolysis that exhibited the highest AUC and lowest false positive rate, meaning the setting with the best capability of distinguishing between true positive and true negative temporal drug outcome combinations without committing too many errors. This empirical evaluation enabled us to determine if the TPD method was suitable for detecting rare temporal adverse drug reaction between statins and rhabdomyolysis.

Since we are unsure what the optimal design choices are for studying statins and rhabdomyolysis using TPD, we evaluated four different settings. It has been previously shown that effect estimates from observational studies are heterogeneous based on the type of database and study design [9, 10], which hence indicates a limitation of the study by Hauben et al. as it only used one study setting [5]. The current study used four timeframes to allow clarity of the rather complex methodology—however future studies can potentially investigate more timeframe options. Nevertheless, these timeframes were chosen as to reflect clinical relevance and ensure generalisability in other settings. The four analytical settings we selected were defined as follows:

- Setting 1: − 180 to − 1 days control period and 1 to 30 days surveillance period (replication of the Hauben study [5])
- Setting 2: − 180 to − 1 days control period and 1 to 360 days surveillance period
- Setting 3: Simultaneous control period (− 180 to − 1 & − 30 to − 1) and 1 to 30 days surveillance period
- Setting 4: Simultaneous control period (− 180 to − 1 & − 30 to − 1) and 1 to 360 days surveillance period

The simultaneous control period allowed for calculations of the ICΔ of multiple candidate windows and picked the lowest because there may be variation in the rate of outcome over the unexposed patient time. This method resulted in an increase in specificity at the expense of a decrease of the sensitivity—as already outlined by Noren et al. [2]. Following this sensitivity analysis, we could then ensure that the best analytical settings were chosen for detecting rare temporal adverse drug reaction between statins and rhabdomyolysis.

#### Development of our LTS software

Our improvement in terms of software development for the calculation of TDPs as compared to the work by Hauben et al. using the THIN database [5] are based on the following. Firstly, we adapted the open-source OHDSI ICTemporalPatternDiscovery package to fasten the chronograph code and extended it to allow for pre-allocation of all by all counts to run faster queries of drug-outcome combinations. The use of open-source software that is applicable to the OMOP common data model also allows for flexibility across different data sources.

**Fig. 1:**
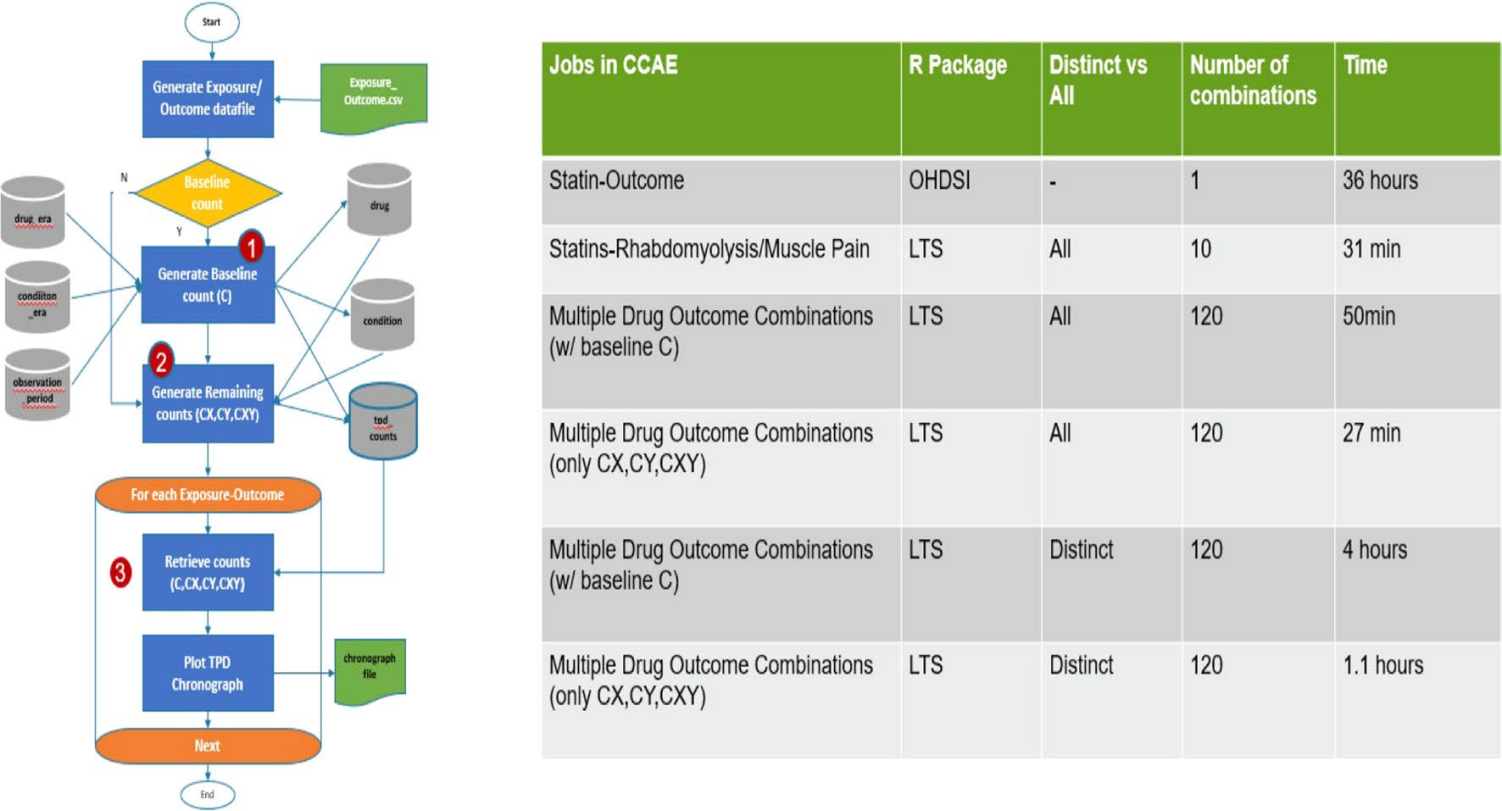
SQL optimisation of R package to calculate TPD with chronographs

Secondly, as the original OHDSI R package was unable to handle the volume of data in CCAE (320 million drug records and 690 million condition records), we developed an SQL optimisation of this R package (Fig. 1). More specifically, to optimise performance time, our R package extracted and stored required and calculated fields from the condition, drug, and observation time tables. It calculated the baseline count (C) using the smaller tables (Step 1). The subsequent calculation steps for the other counts (CX, CY, CXY) could then use these tables. This eliminated multiple joins and calculations within the queries. All counts were stored counts table (Step 2). The stored counts were used to subsequently create multiple chronographs (Step 3). Since the baseline count remained the same regardless of exposure-outcome combinations, a flag was added to skip this calculation saving more time.

Thirdly, we improved the efficiency in generating the chronographs by pre-aggregating all by all counts of drugs and conditions as a reference point for the database. This process ensured that a chronograph from a specific drug-outcome combination can be produced faster. In essence, this creates a reference bank that can be shared with other clinicians and investigators for domain expertise on potential adverse drug events. Our optimisation approach of pre-calculating aggregated data into reference data tables may potentially be useful in optimizing other analytical methods for massive healthcare databases.

## Results

**Fig. 2:**
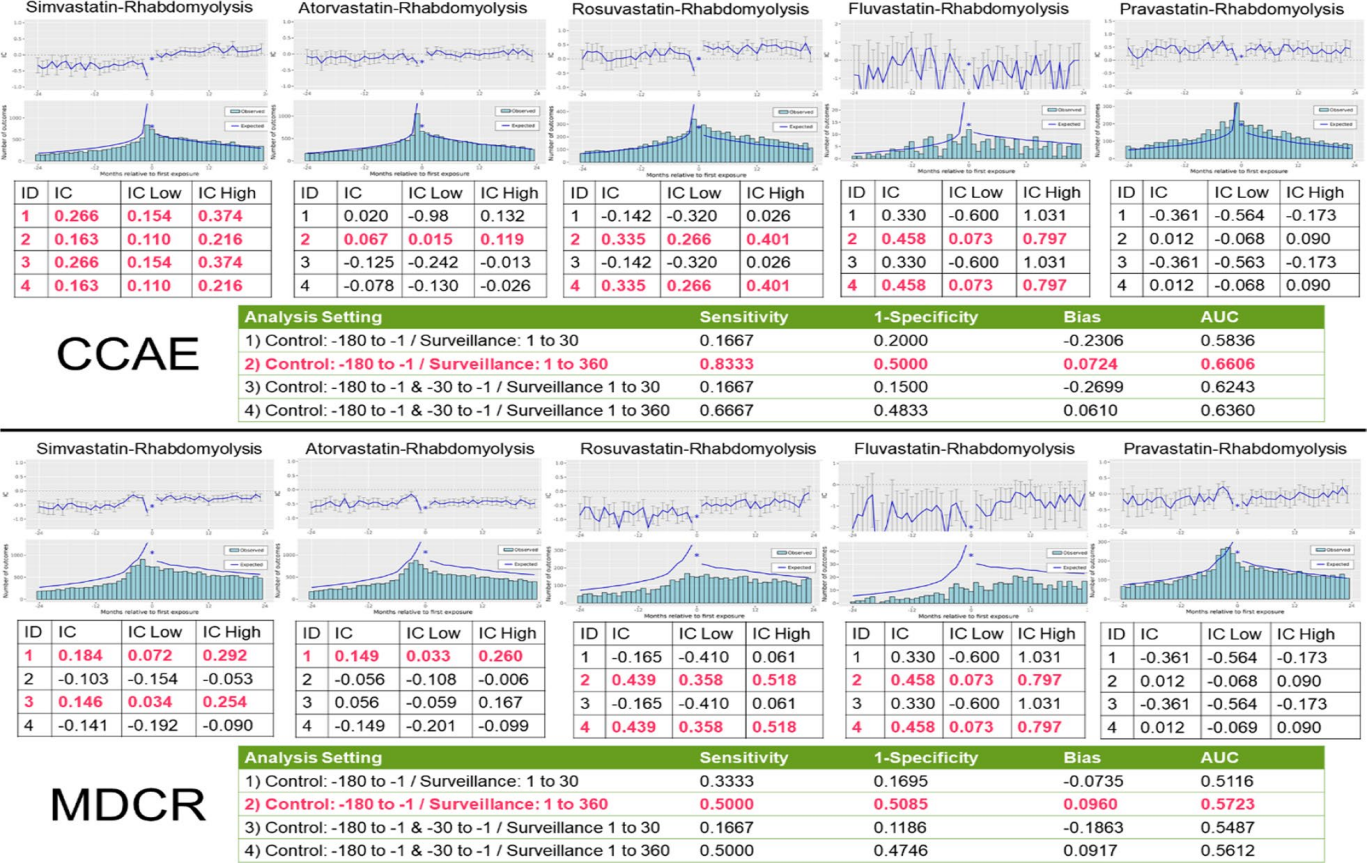
Four analytical settings to detect signal between statins (simvastatin, atorvastatin, rosuvastatin, fluvastatin, pravastatin) and rhabdomyolysis using the CCAE database and MDCR data. Model performance statistics for the different analytical settings are shown for the signal between simvastatin and rhabdomyolysis

Figure 2 shows the chronographs for the five statins studied in relation to rhabdomyolysis using the CCAE and MDCR data. The results replicated the findings in the THIN study [5] as only simvastatin showed a signal with rhabdomyolysis in analytical setting 1. In analytical setting 2, there was a signal for four out of five statins (not for pravastatin), yielding the highest AUC but with a high false positive rate.

**Fig. 3:**
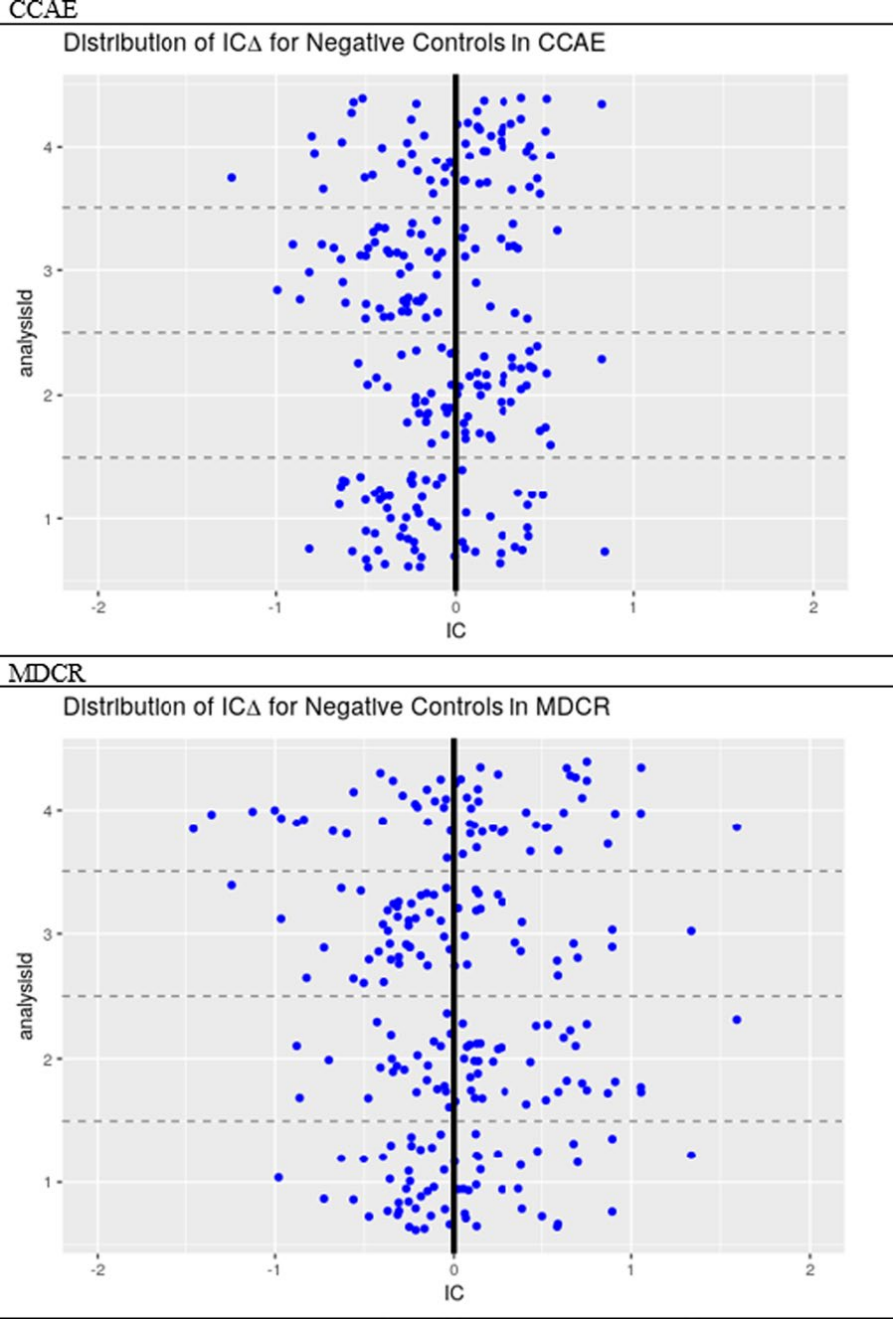
Bias plot using the distribution of the ICΔ for negative controls in the four analytical settings to detect signal between statins (simvastatin, atorvastatin, rosuvastatin, fluvastatin, pravastatin) and rhabdomyolysis

Figure 3 shows the assessment of systematic bias using the negative controls in both the CCAE and MDCR data. There was less of a spread in bias in CCAE than in MDCR. Analyses for Setting 1 and Setting 3 were shifted to the left while analyses for Setting 2 and Setting 4 were shifted to the right.

The improvements of our LTS software package as compared to the existing OHDSI ICTemporalPatternDiscovery are illustrated in Fig. 1. As can been seen in the table of Fig. 1, the computational time decreased substantially, and it became possible to calculate TPDs for various situations simultaneously. For example, the OHDSI package took 36 h to run using just one combination of drug and adverse event. We tested two versions of the LTS package: *All* versus *Distinct*. The first version counted all patients for each control period, while the *Distinct* version only counted exposure date-drug combinations for each control period. An initial test of 10 combinations using the *All* version took 31 min to run. Subsequent tests used 120 combinations and we found that the *Distinct* version took four times longer to run compared to the *All* version. Once the baseline counts were created, both packages were tested again skipping the baseline counts calculations and execution time was reduced to 27 min for the *All* version and 1.1 h for the *Distinct* version.

## Discussion

We were successfully able to replicate the results of the Hauben et al. [5] study of statins and rhabdomyolysis in the US claims dataset mapped to the OMOP common data model. When configuring the TPD analysis to look at statins and rhabdomyolysis in a surveillance window of 1 to 30 days and a control window of 180 to 1 day prior to exposure, only simvastatin had a significant temporal relationship with rhabdomyolysis. Further to the replication of the Hauben study [5], we found the optimal analytical setting for this clinical question to be using a surveillance period of 1 to 360 days and a control period of 180 days to 1 day prior to exposure. Under this setting, four out of the five statins were highlighted with having a temporal association with rhabdomyolysis. It was interesting to note that the lack of a signal for paravastatin is in line with what has been previously observed for the mechanisms of statin intolerance [14, 15].

From a performance perspective, the analytical setting with a surveillance period of 1 to 360 days and a control period of 180 days to 1 day prior to exposure had the highest AUC (0.666) and smallest bias (0.0724), but at the cost of a very high false positive rate (0.5). Following this evaluation, it remains unclear whether TPD is good at detecting rare adverse events. Rhabdomyolysis is a rare condition [16]. This made it a good example to assess the TPD method’s ability to detect rare temporal associations. However, across the four analytical settings and in the two claims databases, it does not seem that TPD is consistent in detecting temporal association of rare events. This reiterates research done of temporal association rules that shows TPD does not show great performance in detecting rare events [17]. Reps suggests that due to the shrinkage applied to TPD, it takes longer for rare ADRs to be signalled, explaining why a longer surveillance window performed better [17]. Further, Arnaud et al. also discuss the limitations of TPD compared to other signal detection methods in longitudinal observational databases [1]. One major limitation is that it does not provide risk estimates; only flagging if a temporal association exists. Second, the TPD method does a poor job of controlling for time-varying confounding despite leveraging an external cohort in its design. Typically, the external cohort is left open-ended to include all available drugs to improve computational efficiency for the purpose of conducting an initial database scan. However, one should include drugs of similar indication as a contrast to control for time-varying confounding; this comes at the cost of poor computational efficiency [2]. Perhaps our ability to detect more signal, at a higher false positive rate, in a longer surveillance window could have been impacted by this limitation in the method.

A limitation of our analysis was the selection of positive controls. Only the five statins were used as positive controls, since it was difficult to identify drugs that had a known (or suspected) positive association with rhabdomyolysis. A potential solution to this issue would be to use the OHDSI R Package MethodEvaluation which contains a function to create synthetic positive controls to help calculate operating characteristics. However, this function requires risk estimates to synthetically create positive controls and one would need to tailor this to work with IC values generated from the TPD method. A future enhancement to our method would be to include synthetic positive controls for TPD. While our performance evaluation may have been limited, it is vital that one evaluates the operating characteristics of these signal detection methods on longitudinal observational databases. Following the suggestions outlined by the book of OHDSI are important for conducting signal detection [13].

Through this project we also added a technical enhancement to query marginal counts for drugs and outcomes needed to build chronographs for the TPD method. An advantage for using TPD is the graphical component of the chronograph, allowing domain experts to validate potential temporal signals. The open source ICTemporalPatternDiscovery R package from the OHDSI community can create a chronograph, but queries take a very long time. Our enhancement was to pre-allocate marginal drug and outcome counts, reserving them in our database management system to accelerate chronograph queries. While this enhancement is helpful for TPD, the concept of pre-allocation may prove useful for other database methods in pharmacoepidemiology allowing for rapid queries to scan for potential ADRs.

## Conclusions

The TPD method is a useful signal detection tool that provides a single statistic on temporal association and a graphical depiction of the temporal pattern of the drug outcome combination. It remains unclear if the method works well for rare adverse events, but it has been shown to be a useful risk identification tool for longitudinal observational databases. Future work should compare the performance of TPD with other pharmacoepidemiology methods and mining techniques of signal detection. In addition, it would be worth investigating the relative TPD performance characteristics using a variety of observational data sources.

## Data Availability

R package is made publicly available on the OHDSI github.

## Abbreviations

AUC: Area under the curve
CCAE: Commercial Claims and Encounters
IC: Information Component
MDCR: Medicare Supplemental
ODHSI: Observational Health Data Sciences and Informatics
OMOP: Observational Medical Outcomes Partnership
SQL: Structured Query Language
THIN: The Health Improvement Network
TPD: Temporal pattern discovery
